# Arrhythmias and Heart Failure in Pregnancy: A Dialogue on Multidisciplinary Collaboration

**DOI:** 10.3390/jcdd9070199

**Published:** 2022-06-24

**Authors:** Kamala P. Tamirisa, Cicely Dye, Rachel M. Bond, Lisa M. Hollier, Karolina Marinescu, Marmar Vaseghi, Andrea M. Russo, Martha Gulati, Annabelle Santos Volgman

**Affiliations:** 1 Texas Cardiac Arrhythmia Institute, Dallas, TX 78704, USA; 2 Division of Cardiology, Rush University Medical Center, Chicago, IL 60612, USA; cicely_a_dye@rush.edu (C.D.); karolina_marinescu@rush.edu (K.M.); annabelle_volgman@rush.edu (A.S.V.); 3 Division of Cardiology, Dignity Health, Chandler, AZ 85224, USA; drrachelmbond@gmail.com; 4 Department of Obstetrics & Gynecology, Baylor College of Medicine, Houston, TX 77030, USA; lisaholier1@gmail.com; 5 Division of Cardiology, UCLA Cardiac Arrhythmia Center, Los Angeles, CA 90095, USA; mvaseghi@mednet.ucla.edu; 6 Division of Cardiology, Cooper University Hospital, Camden, NJ 08103, USA; russo-andrea@cooperhealth.edu; 7 Barbra Streisand Women’s Heart Center, Smidt Heart Institute, Cedars-Sinai Medical Center, Los Angeles, CA 90048, USA; martha.gulati@gmail.com

**Keywords:** collaborative, cardio-obstetrics, women, CVD

## Abstract

The prevalence of CVD in pregnant people is estimated to be around 1 to 4%, and it is imperative that clinicians that care for obstetric patients can promptly and accurately diagnose and manage common cardiovascular conditions as well as understand when to promptly refer to a high-risk obstetrics team for a multidisciplinary approach for managing more complex patients. In pregnant patients with CVD, arrhythmias and heart failure (HF) are the most common complications that arise. The difficulty in the management of these patients arises from variable degrees of severity of both arrhythmia and heart failure presentation. For example, arrhythmia-based complications in pregnancy can range from isolated premature ventricular contractions to life-threatening arrhythmias such as sustained ventricular tachycardia. HF also has variable manifestations in pregnant patients ranging from mild left ventricular impairment to patients with advanced heart failure with acute decompensated HF. In high-risk patients, a collaboration between the general obstetrics, maternal-fetal medicine, and cardiovascular teams (which may include cardio-obstetrics, electrophysiology, adult congenital, or advanced HF)—physicians, nurses and allied professionals—can provide the multidisciplinary approach necessary to properly risk-stratify these women and provide appropriate management to improve outcomes.

## 1. Introduction

An increasing maternal morbidity and mortality has been observed in the United States over the past two decades despite advances in obstetric medicine. Although the causes for this increase are multi-factorial, the simultaneous rising incidence of cardiovascular disease (CVD) is likely to be a major contributor to maternal and fetal complications and mortality in the United States [1]. According to the Center for Disease Control (CDC), from 2014 to 2017, deaths related to cardiomyopathies and other cardiovascular conditions accounted for approximately 27% of pregnancy-related deaths in the United States [2]. 

The prevalence of CVD in pregnant people is estimated to be around 1 to 4% [3], and it is imperative that clinicians that care for obstetric patients can promptly and accurately diagnose and manage common cardiovascular conditions as well as understand when to promptly refer to a high-risk obstetrics team for a multidisciplinary approach for managing more complex patients [4]. In pregnant patients with CVD, arrhythmias and heart failure (HF) are the most common complications that arise [5]. The difficulty in the management of these patients arises from the variable degrees of severity of both arrhythmia and heart failure presentation. For example, arrhythmia-based complications in pregnancy can range from isolated premature ventricular contractions to life-threatening arrhythmias such as sustained ventricular tachycardia. HF also has variable manifestations in pregnant patients ranging from mild left ventricular impairment to patients with advanced heart failure with acute decompensated HF. 

In high-risk patients, a collaboration between the general obstetrics, maternal–fetal medicine, and cardiovascular teams (which may include cardio-obstetrics, electrophysiology, adult congenital, or advanced HF)—physicians, nurses and allied professionals—can provide the multidisciplinary approach necessary to properly risk-stratify these women and provide appropriate the management to improve outcomes. In lower-risk patients, consultation with the appropriate specialist can help guide management regarding the appropriate treatment. Even when the multidisciplinary team agrees upon the management plan, the treatment of arrhythmias and HF in pregnancy can still prove difficult due to the potential for the teratogenicity of some of the medications commonly used to treat HF and arrhythmias. Clinicians need to know when an intervention during pregnancy is appropriate versus when it is safe to pursue a more conservative approach such as watchful waiting or reassurance. A collaborative cardio-obstetrics team approach can be helpful not only in the medical management of patients, but in setting patient expectation and providing education. 

## 2. Arrhythmias in Pregnancy

The prevalence of arrhythmias in pregnancy has been increasing over the past two decades in the U.S. [6]. This rise is attributed to the prevalence of CVD associated with increasing maternal age and other comorbidities such as hypertension, obesity, and diabetes mellitus [7,8,9]. Premature atrial and ventricular contractions are common due to hormonal and volume changes; apart from these, the most common maternal arrhythmias in pregnancy in the order of observed prevalence are supraventricular arrhythmias, atrial fibrillation, and ventricular arrhythmias [6]. Symptoms include palpitations, presyncope, chest pain, and syncope, especially in the setting of ventricular arrhythmias. 

### 2.1. Supraventricular Tachycardia

Supraventricular tachycardia (SVT) is the most common type of arrhythmia in pregnancy [6]. In women with normal hearts, SVT is more likely due to atrioventricular nodal reentrant and/or atrioventricular reentrant tachycardias. Given the involvement of the atrioventricular node in the etiology and mechanism of these arrhythmias, vagal maneuvers are effective and the first-line treatment strategy [7]. Adenosine can be safely used in pregnancy and is the medication of choice for the termination of maternal SVT [10]. For the treatment of recurrent arrhythmias, beta-blocker therapy (metoprolol and propranolol) is the first line. Calcium channel blockers (verapamil and diltiazem) are the second line of management. Digoxin is a safe treatment option for rate control [7]. 

Atrioventricular nodal blocking agents alone, especially digoxin, must be avoided in patients with evidence of pre-excitation and SVT [11]. The clinical signs of digoxin toxicity and its levels should be monitored closely, as the levels of digoxin during pregnancy can be unreliable and compromised due to the circulation of digoxin-like fragments [12]. Flecainide can be safely used to treat SVT in structurally normal hearts in both the mother and fetus, though rarely, neonatal toxicity can occur [13]. In the setting of hemodynamic compromise, as with other arrhythmias, cardioversion can be safely performed in pregnancy, and defibrillation pads should be placed away from the gravid uterus [7]. Successful ablation of SVT has been reported during pregnancy with minimal fluoroscopy [14,15,16]; however, deferring ablation to the postpartum period is preferred. As in other arrhythmias, ablation prior to conception is recommended in patients with a history of known recurrent and symptomatic SVT prior to pregnancy. 

### 2.2. Atrial Flutter and Fibrillation

#### 2.2.1. Epidemiology and Risk Factors

While atrial fibrillation (AF) was historically an uncommon arrhythmia during pregnancy, the prevalence has increased in more recent years. It is now one of the most frequently occurring arrhythmias during pregnancy, particularly in women with underlying structural heart disease [6,17]. AF was infrequent and reported in only two of 100,000 pregnancies between 1992 and 2000 [18]. In a nationwide inpatient sample from 2000 to 2012, the overall frequency of AF was 27 per 100,000, and atrial flutter (AFL) was 4 per 100,000 pregnancy-related hospitalizations [6]. The number of pregnancy-related hospitalizations with AF increased by 111% from 2000 to 2012, from 18 per 100,000 to 35 per 100,000 (*p* < 0.001). AF is more common in women of advanced maternal age [6,19]. It is also more common in women with underlying structural heart disease including congenital heart disease [17,20]. The increased prevalence of AF is likely to be related to advanced maternal age, increased risk factors (such as hypertension, diabetes mellitus, obesity, obstructive sleep apnea), and congenital heart disease during pregnancy. AFL is uncommon in women without congenital heart disease [17]. 

AF more commonly develops during the end of the second trimester or the third trimester [19,21]. In women with preexisting AF/AFL, exacerbation of arrhythmia during pregnancy is common, with a recurrence rate of 52% [22]. In a large prospective cohort study of initially healthy women, an increasing number of pregnancies was associated with a subsequent increased risk of developing AF after adjusting for age and other cardiovascular and AF risk factors [23]. A systematic review confirmed that an increasing number of pregnancies was associated with an increased risk of AF, which had a dose–response relationship [24]. It is hypothesized that repeated exposure to metabolic, physiological, and hormonal changes during pregnancy may predispose to AF.

AF during pregnancy may result in poor maternal and fetal outcomes. Adverse maternal outcomes associated with AF include a high risk for preeclampsia and maternal congestive heart failure [20]. Maternal mortality is also higher in women with AF/AFL compared to those without AF/AFL [6,21].

#### 2.2.2. Treatment

While pregnancy is associated with a hypercoagulable state, there is limited data regarding the risk of stroke during pregnancy. The European AF Guidelines recommend that the same risk assessment be used in pregnant and non-pregnant individuals [25]. As in all patients, the risks and benefits of anticoagulation need to be assessed, considering both maternal and fetal risks in the pregnant patient. Direct oral anticoagulants have not been adequately evaluated for safety and should not be used during pregnancy. Instead, therapeutic anticoagulation with heparin or vitamin K antagonists according to the stage of pregnancy is recommended [13,25].

For patients with mechanical prosthetic valves who require <5 mg/day of warfarin, the European guidelines favor oral anticoagulants throughout pregnancy with a change to the unfractionated heparin before delivery. However, not all practitioners may feel comfortable using warfarin during pregnancy, and dosing requirements may not remain stable over time. For patients with prosthetic valves who require higher doses, switching to low molecular weight heparin during the first trimester with strict anti-Xa monitoring is recommended with a change to the unfractionated heparin before delivery [26].

With respect to the acute treatment of AF during pregnancy, the European guidelines recommend immediate direct current cardioversion for hemodynamically unstable AF as a class I recommendation [25]. This treatment can be performed safely without compromising fetal blood flow. Intravenous beta-blockers are recommended for acute rate control. For long-term treatment, beta-1 selective blockers (e.g., metoprolol) are considered as first choice (class I recommendation), while digoxin and verapamil could be considered if beta-blockers fail (class IIa recommendation) [25]. Atenolol should be avoided due to intrauterine growth restriction. Rhythm control during pregnancy is desirable. Flecainide, propafenone, or sotalol should be considered to prevent recurrent AF (class IIa recommendation) [25]. Amiodarone should be avoided whenever possible as it may result in fetal thyroid toxicity. It should be noted that since clinical data are limited in pregnancy, guidelines for AF management in pregnancy are mostly based on expert opinion (Level C evidence). As in a non-pregnant population, drug selection should be based on the presence or absence of underlying structural heart disease. While there have been reports of catheter ablation performed during pregnancy [27], the European guidelines state that “AF catheter ablation has no role during pregnancy [25]”.

### 2.3. Ventricular Tachycardia

The incidence of ventricular arrhythmias has been increasing in the United States [6], and the frequency of these arrhythmias can be exacerbated by the physiological changes of pregnancy. Incidence of VT is greatest in pregnant patients with congenital heart disease, occurring in up to 27% [22]. Outside of congenital heart disease, VT can occur in the setting of cardiomyopathy and spontaneous coronary artery dissection and spasm. Acute management of ventricular tachycardia in hemodynamically stable patients includes beta-blocker therapy (metoprolol and propranolol) followed by lidocaine [7]. The use of quinidine and procainamide depends on the presence of underlying heart disease. These agents should be avoided in the setting of coronary artery disease or ischemia, as in coronary artery dissection [28]. In patients without structural heart disease and ventricular arrhythmias, flecainide, sotalol, or quinidine can be safely used to prevent recurrences. Verapamil can be safely administered in the setting of fascicular VT to terminate arrhythmias and can be used to prevent recurrences [29]. Although amiodarone is generally to be avoided during pregnancy, short-duration amiodarone use for the treatment of refractory fetal arrhythmias for a duration of one to 15 weeks did not demonstrate any fetal adverse events [30]. 

Ventricular arrhythmias are the most concerning manifestation of inherited arrhythmia syndromes during pregnancy. For long QT syndrome, especially type I and II, and catecholaminergic polymorphic VT, beta-blocker therapy with metoprolol and propranolol should be continued throughout pregnancy [7]. In the setting of polymorphic VT due to long QT syndrome or other causes, IV magnesium can be safely administered in pregnancy [31]. Although Brugada syndrome has a male predominance, quinidine can be used to control ventricular arrhythmias during pregnancy as in pre-pregnancy [13].

As in other arrhythmias, hemodynamically unstable patients with ventricular tachycardia warrant cardioversion, and energy levels between 50 and 400 J have been reportedly used without adverse effects [7,32,33,34]. Although catheter ablation of ventricular tachycardia during pregnancy has been safely and successfully performed with minimal fluoroscopy [35,36,37], deferring ablation to the postpartum period is preferred. 

### 2.4. Cardiac Arrest

Cardiac arrest during pregnancy is rare. More common causes include hemorrhage, anesthetic complications, amniotic fluid embolism, and cardiovascular causes including myocardial infarction, aortic dissection, and pulmonary embolus should be considered [38]. Importantly, cardiopulmonary resuscitation protocols including medication doses, frequency of chest compressions, and defibrillation in pregnancy are similar to non-pregnancy, with the exception of lateral displacement of the uterus after 20 weeks of gestation and maternal survival is prioritized [39]. If initial basic life support and advanced cardiac life support interventions fail to restore maternal circulation within 4 min of cardiac arrest, peri-mortem delivery is advised, provided the uterus is ≥20 weeks in size [39].

## 3. Heart Failure and Pregnancy

During pregnancy, HF can be a preexisting condition or occur de novo. Preexisting cardiomyopathies (CMP) can be classified according to left ventricular systolic function: HF with reduced ejection fraction (EF) (HFrEF), HF with mid-range ejection fraction (HFmrEF), and HF with preserved ejection fraction (HFpEF), where the EF is <40%, 40–50%, or >50%, respectively. HF can also be classified by etiology such as ischemic, hypertensive, valvular, dilated, restrictive (e.g., hypertrophic cardiomyopathy (HCM)), infiltrative, and arrhythmia-related such as arrhythmogenic right ventricular cardiomyopathy (ARVC) [40,41]. Pregnancy-associated or peripartum cardiomyopathy (PPCM) is diagnosed between 36 weeks of gestation and up to five months following delivery [2].

HF poses several challenges during the peripartum period, whether chronic or pregnancy-associated. The physiologic changes in pregnancy, namely the 10–30 bpm increase in heart rate, decrease in systemic vascular resistance, and 30–50% increase in blood volume and cardiac output, can result in decompensation [42,43,44]. HF symptoms may sometimes be difficult to differentiate from those related to physiologic changes of pregnancy, making the condition more challenging to evaluate. Furthermore, there are limitations in the medical therapy choices during pregnancy, and adjustments are needed in preparation for a pregnancy in women with a preexisting cardiomyopathy. 

### 3.1. Symptoms

It is common for patients with uncomplicated pregnancies to experience peripheral edema, dyspnea, fatigue, and decreased exercise tolerance. An evaluation may be warranted when symptoms are extreme, particularly in individuals with a prior diagnosis of HF or predisposing conditions. The CARPREG II risk score is a useful tool to evaluate patient symptoms and to determine the frequency of antenatal care visits [5]. The score includes ten predictors: prior cardiac events or arrhythmias (3 points), poor functional class (NYHA III-IV) or cyanosis (3 points), mechanical valves (3 points), ventricular dysfunction with EF < 55% (2 points), high-risk valve disease or left ventricular outflow tract obstruction (2 points), pulmonary hypertension (2 points), coronary artery disease (2 points), high-risk aortopathy with aortic dimension > 45 mm (2 points), no prior cardiac intervention (1 point), and late pregnancy assessment (1 point). The risk of cardiac events increases with a higher score—0–1 point (5%), 2 points (10%), 3 points (15%), 4 points (22%), and >4 points (41%).

### 3.2. Management

Patients with structural heart disease require close monitoring and medical management. The main goal of treatment is to improve the symptoms and optimize the hemodynamics. Certain types of HF encountered in pregnancy are highlighted below for specific recommendations. Regardless of etiology, the initial evaluation should include an assessment of respiratory status and signs of fluid retention, utilizing diuretic therapy as needed. 

### 3.3. HFrEF/Dilated CMP/PPCM

In addition to symptom management, treatment goals should also include optimizing hemodynamics and improving left ventricular systolic function. However, the initiation and up-titration of guideline-directed medical therapy are limited during the peripartum period. As angiotensin-converting enzyme inhibitors, angiotensin II receptor blockers, angiotensin receptor-neprilysin inhibitors, and mineralocorticoid antagonists are teratogenic, and the use of these agents is contraindicated during pregnancy. Beta-blockers may be initiated and should be continued. For symptomatic patients, diuretics can improve congestion. If symptoms persist despite optimizing volume status, digoxin can be considered. Advanced therapies such as inotropic and vasoactive agents should be considered as temporary or during mechanical circulatory support for unstable patients with acute or acute-on-chronic HF [45,46,47].

### 3.4. Postpartum Cardiomyopathy (PPCM)

PPCM is a form of idiopathic cardiomyopathy (with a reduced left ventricular ejection fraction of <45% with or without left ventricular dilatation) seen toward the end of pregnancy or up to 5 months post-partum [48,49]. Several risk factors that contribute to increased incidence of PPCM include preeclampsia, hypertension, advanced age at pregnancy (>30 years), and African-American race [50,51,52]. Diagnosis may be delayed because the symptoms can mimic the findings of an uncomplicated pregnancy. Underlying pathophysiology is probably multifactorial—viral, vascular, and autoimmune processes [53,54,55]. The role of genetic and epigenetic factors involving TTN truncating variants have been suggested to be similar in dilated cardiomyopathy patients [56]. PPCM is associated with a higher risk of cardiopulmonary arrest, pulmonary edema, thromboembolic (left ventricular thrombus was noted in 10–17% echocardiograms), and cerebrovascular events [57,58]. One year mortality rates in PPCM ranged from 4% in the IPAC study to around 11% in a predominantly African-American female population [59,60]. Sudden cardiac death due to VT and VF have been reported in observational and small prospective studies [61,62,63]. Poor prognostic indicators include low left ventricular ejection fraction (<30%), obesity, African-American ethnicity, and left ventricular thrombus [64]. Most women recover within 3–6 months and at times delayed recovery until 2 years is seen. Treatment includes guideline-directed optimal medical management (diuretics, metoprolol, hydralazine/nitrates, digoxin, and low molecular weight heparin) such as aa wearable or implantable defibrillator [59]. While most women recover within 3–6 months, delayed recovery is noted for up to 2 years [59]. Wearable defibrillators may be considered for women with new-onset PPCM as a bridge to recovery or implantable defibrillator [61,62]. Bromocriptine as a treatment option was driven by the concept of inhibiting prolactin secretion to prevent PPCM seen in mice models. African studies and the German observational registry have shown improved LVEF and improved mortality in women with PPCM [65,66]. Currently, bromocriptine is not approved as a definitive therapy for PPCM. The implications of the inability to breastfeed and the need for concomitant anticoagulation needs to be discussed with the mother [47]. Subsequent pregnancies and discussion of the risk calls for shared decision making and pre-pregnancy LVEF is the strongest predictor of outcomes [49]. 

### 3.5. Restrictive Cardiomyopathy (RCM)

In RCM, the systolic function is preserved; however, LV hypertrophy results in impaired relaxation. Beta-blockers and calcium channel blockers are first-line therapy to reduce the heart rate and increase the diastolic filling time. In the case of HCM, peripheral vasodilators should be avoided as these can worsen the left ventricular outflow tract gradient and promote decompensation. If arrhythmias are refractory to beta-blockers or calcium channel blockers, notably in the case of HCM and ARVC, Class IC agents such as flecainide or propafenone can be used [47].

### 3.6. Valvular Heart Disease

Women with severe mitral and aortic valve disease are advised against pregnancy. Women with less severe valve disease who choose to become pregnant are advised to be followed closely for signs and symptoms of arrhythmias and heart failure. Medical management with drugs considered safe in pregnancy can be used but in patients with refractory symptoms, balloon valvuloplasty, or surgical valve replacement may be offered [3].

The summary of the arrhythmias and heart failure management during pregnancy is shown in Table 1. 

### 3.7. Pulmonary Hypertension

Pregnancy is not advised for women with pulmonary arterial hypertension (PAH) [3]. However, in 2007, the European Society of Cardiology began a European Registry on Pregnancy and Cardiac Disease (ROPAC). There were 151 women in a subgroup with pulmonary hypertension (PH). Pregnancy carried a substantial risk and commonly resulted in heart failure despite therapeutic options. In the small number of patients with idiopathic PAH (3/7 or 43%), it led to mortality. The risk was lower in other causes of PAH such as congenital heart disease and left heart disease but there were significant differences in the maternal and fetal outcomes of women. Therefore, women should be counselled before conception about the short- and long-term risk to their health, and also about the poor outcomes for their offspring [49]. 

### 3.8. Pathologies That Are Contraindications to Pregnancy Due to Arrhythmias and Heart Failure

Certain cardiac pathologies preclude safe and optimal pregnancy outcomes. These pathologies are listed with potential adverse cardiac events in the table [47,67,68,69,70,71,72,73,74,75,76,77] (Table 2).

### 3.9. Cardiovascular Medications during Pregnancy

Former Food and Drug Administration’s ABCDX categories are not used solely for categorizing medications. Currently, these medications are listed according to the clinical indications (Table 3).

## 4. Current Gaps in CVD Care

### Growing Concern

The United States is the only industrialized country where maternal mortality is rising, despite as many as sixty percent of the seven-hundred pregnancy-related deaths each year being preventable [1]. 

The national trend of increasing maternal age has led to more women entering pregnancy with chronic medical conditions and greater cardio metabolic risk factors such as obesity, high blood pressure, and diabetes. Women who develop pregnancy complications such as preeclampsia, hypertensive disorders of pregnancy, premature labor, and gestational diabetes are at a greater risk for adverse pregnancy outcomes and increased risk for CVD following pregnancy [78].

Disparities in maternal health are notable, with Black women facing 3.2 times higher risk of pregnancy-related death. Native-American and Native-Alaskan women face a 2.5 times higher risk than non-Hispanic White women [1]. These disparities persist even among college-educated Black women and are greatly driven by psychosocial stressors including structural racism through the conceptual framework of the “superwoman schema [79]”.

This “biology of adversity” framework of stresses with the history of stereotyping, oppression, disappointment, and abuse, along with spiritual values and foremother influences, among Black women interface with the neurobiological axis. This combination results in the upregulation of the sympathetic nervous system and hypothalamic axis and inflammatory dysregulation, resulting in untoward maternal and nonmaternal health outcomes [80]. These adverse changes can lead to exacerbations in both HF and arrhythmias.

The elements of these psychosocial stressors, compounded by pregnancy as a natural stress test and window to future health, impact these women’s cognitive and cardiovascular health prematurely, leading to the maternal health crisis, which disproportionately affects Black women.

## 5. Building Multidisciplinary Collaboration and Care Teams (MDCCT)

### 5.1. Role of MDCCT to Close Gaps in Care

In response to the longstanding Black maternal health crisis, the Association of Black Cardiologists, Inc. convened a Black Maternal Heart Health Roundtable in June 2020 with a subsequent publication that outlined unifying solutions to improve health outcomes for Black women across the age continuum [81]. Multidisciplinary collaborations with obstetrics, maternal–fetal medicine, cardiology, primary care clinicians, midwives, doulas, and pediatricians were at the forefront of the discussion, see Table 4. These specialties were suggested to be included in the cardio-obstetrics team to acknowledge the role that unique risk factors play in disparities in maternal care and outcomes and, most importantly, to ensure that uninterrupted care is provided throughout the entire maternal health continuum from preconception to postpartum. Beyond this, the novel approach to including community leaders has been shown to be effective in other aspects of CVD management and care [82]. The suggestion of their inclusion in the maternal care team as a way to bridge communication and improve the distrust in these marginalized communities was also deemed to be beneficial.

### 5.2. Practical Aspects to Building MDCCT and Policies

**Figure 1:**
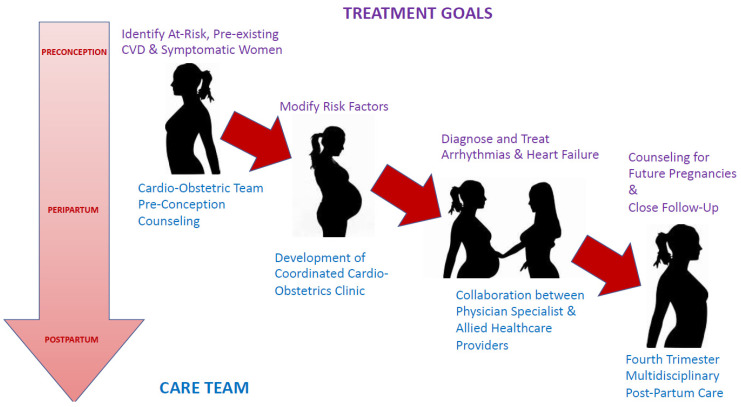
Care Team and Treatment Goals.

As shown in the Figure 1, a multidisciplinary care team starts during the pre-conception phase and is required throughout the pregnancy and postpartum phase, especially in women with HF and arrhythmias. Coordinated cardio-obstetrics clinics have decreased adverse cardiac complications during and after pregnancy [5]. The members of the cardio-obstetrics team (also referred to as the pregnancy heart team) may vary depending on the patient’s specific needs. However, typically teams include the following when HF or significant arrhythmias are present: cardio-obstetrics, cardiovascular medicine, maternal–fetal medicine, obstetrics, primary care, genetics, nursing, pharmacy, social work, and other specialists including anesthesiologists, HF cardiologists, electrophysiologists, and allied professionals [83,84]. A neonatologist is a vital team addition to discuss fetal/neonatal risks when preterm delivery may be indicated see (Figure 1). 

The needs of the patient and institutional resources may vary, so the collaboration may occur through co-located clinics, multidisciplinary care conferences, or independent inpatient or outpatient service lines. It is essential to develop physical and virtual patient care networks, allowing women to receive the level of care they need close to their homes whenever possible [85]. 

Ideally, the cardio-obstetrics team would evaluate women with CVD desiring pregnancy prior to conception to discuss the maternal/fetal risks, optimize maternal health status, review potential medication adjustments, and educate about surveillance during and after pregnancy. Evaluation early in pregnancy is beneficial for risk stratification. Regular follow-up visits and team meetings throughout pregnancy are essential to facilitate patient-centered decisions and create a detailed, individualized, and regularly updated management plan, particularly for the coordination of care for delivery. The delivery plan should be created and recorded in the medical record by the beginning of the third trimester. It should be readily accessible for all members of the health care team. 

The postpartum period is a time of increased risk for the gravida with CVD, particularly for those with HF. Among the CVD-related causes of maternal mortality, peripartum cardiomyopathy is the leading cause of late postpartum death [8,9,86]. Creating a coordinated, interdisciplinary plan for discharge and subsequent postpartum assessment and management is critical for ongoing safety. Individualized plans should address patient-specific warning signs of worsening CVD, early postpartum follow-up, considerations regarding breastfeeding, emotional support, future pregnancy intentions, commensurate contraceptive needs, and long-term cardiovascular follow-up [83].

The American College of Obstetricians and Gynecologists recommends a postpartum follow-up visit (early postpartum visit) with either the primary care provider or cardiologist within 7–10 days of delivery for women with hypertensive disorders or 7–14 days of delivery for women with CVD [84].

There are multiple barriers to optimized cardio-obstetric care including (1) delays in the identification and diagnosis of women with acquired CVD; (2) barriers to accessing high-quality care; and (3) limited insurance coverage for many individuals. Data from maternal mortality review committees have found that nearly 70% of cardiovascular and coronary deaths are preventable. Provider-related factors, most commonly delayed diagnosis and appropriate treatment, contribute to a significant proportion of maternal deaths. Multidisciplinary education about pregnancy and heart disease is critical to optimizing care. 

With Black women, the greatest risk group, systemic discrimination and implicit racial bias are important barriers to high-quality care [81]. This is best highlighted by the fact that educational attainment, income, and employment do not protect Black women from the staggering rates of poor outcomes [87]. Other barriers include transportation, childcare, and rurality. Policy reform at the local, state, and federal levels is necessary to address racism and the social drivers of health. Finally, optimized cardio-obstetric care is complicated by the lack of insurance coverage for many non-pregnant individuals, sudden loss of Medicaid coverage after delivery, and current bundled payment models that disincentivize multiple postpartum visits. Payment reforms including Medicaid expansion in the remaining states and Medicaid extensions to 12 months postpartum are solutions to optimize health care in the critical preconception and postpartum periods [88]. In April 2021, Illinois became the first state to provide full Medicaid benefit coverage for mothers during the postpartum period for a full year and several states have since followed suit [89]. 

## 6. Conclusions

The management of arrhythmias and HF in pregnancy most often go hand in hand, as these two cardiovascular conditions co-exist, one contributing to the other and vice versa. Successful management of these patients requires collaboration between the service lines and all stakeholders, with shared decision-making with the patient at the center of the paradigm. This plan includes transfer care coordinators for those at significantly high risk for cardiac compromise to centers with advanced HF teams, cardiovascular surgery, critical care, cardiac electrophysiologists, and neonatal care. Education and understanding of the social determinants of health (SDOH) along with policy reforms are imperative in this multidisciplinary care model, along with affordable access to expertise.

## Figures and Tables

**Table 1:** Arrhythmias and heart failure management in pregnancy.

	Arrhythmias Management in Pregnancy
Supraventricular Tachycardia (SVT)	First-line treatment: Vagal maneuver. If ongoing arrhythmias: 1. Beta blockers (e.g., Metoprolol, Propranolol). 2. Calcium channel blocker (e.g., Verapamil, Diltiazem) 3. Digoxin [with monitoring for digoxin toxicity] 4. Anti-arrhythmic agents (e.g., Flecainide) If medically resistant: 1. Direct Current Cardioversion (DCCV) [especially if hemodynamic compromise] 2. SVT ablation with minimal/zero fluoroscopy
Atrial Flutter & Atrial Fibrillation	1. Anticoagulation: Use the same risk assessment for cardio embolic events. a. Mechanical prosthetic valves with <5 mg/day of Warfarin dose → can continue anticoagulants throughout pregnancy with a change to unfractionated heparin before delivery (better to avoid warfarin until 12 weeks) b. Mechanical prosthetic valves with higher doses, switching to LMWH during the first trimester with strict anti-X1 monitoring is recommended with a change to unfractionated heparin before delivery. 2. Acute rate control a. Intravenous beta-blockers (e.g., Metoprolol) b. Intravenous Verapamil, Digoxin c. If medically-resistant to rate control (especially if hemodynamic instability) consider DCCV 3. Long-term treatment a. Beta-1 selective blockers (e.g., Metoprolol) (class Ia) b. If fails, Verapamil and/or Digoxin (class IIa) c. Rhythm control with flecainide, propafenone or sotalol (class IIa)
	**Arrhythmias Management in Pregnancy**
Ventricular Tachycar-dia	Acute management (hemodynamically stable) 1. Beta-blocker (e.g. Metoprolol and Propranolol) 2. Lidocaine 3. Verapamil (first line for fascicular VT) 4. IV magnesium (in the setting of polymorphic VT due to Long QT syndrome) If ongoing arrhythmia: 1. Quinidine and/or procainamide (excluding underlying heart disease) 2. Flecainide, sotalol or quinidine (to prevent recurrence) 3. Amiodarone (in the shortest possible duration for the use of refractory fetal arrhythmias for duration of one to 15 weeks). Acute management (hemodynamically unstable) Cardioversion with energy levels between 50 to 400 Joules.
Cardiac Arrest	Cardiopulmonary resuscitation protocols, including medical doses, frequency of chest compressions, and defibrillation in pregnancy are similar to non-pregnancy, with the exception of lateral displacement of the uterus after 20 weeks of gestation.
	**Heart Failure Management in Pregnancy**
Heart failure with reduced ejection fraction (HFrEF)	e.g., Dilated cardiomyopathy; Peripartum cardiomyopathy. Guideline-line directed medical therapy (GDMT) contraindicated due to teratogenic nature include: angiotensin-converting enzyme inhibitors, angiotensin II receptor blockers, angiotensin receptor-neprilysin inhibitors and mineralocorticoid antagonists. GDMT safe to use with pregnancy: 1. Beta-blockers Symptomatic management: 1. Diuretics 2. Digoxin 3. Advance therapies such as inotropic and vasoactive agents should be considered temporary or during mechanical circulatory support for unstable patients with acute or acute on chronic HF.
Heart failure with preserved ejection fraction (HFpEF)	e.g., Restrictive Cardiomyopathy; Hypertrophic cardiomyopathy, ARVC. First-line: Beta blockers and/or calcium channel blockers to reduce the heart rate and increase diastolic filling time (Peripheral vasodilators should be avoided to prevent the worsening of the left ventricular outflow tract gradient)

**Table 2:** Pathologies that are contraindications to pregnancy due to high risk of arrhythmias and heart failure.

Cardiomyopathies	Medical Recommendations	High Risk States for Pregnancy	Adverse Cardiac Events
Dilated Cardiomyopathy: post-viral/myocarditis; inflammatory disease; tachycardia mediated; storage diseases, toxin induced, Takotsubo (stress-mediated), Post-partum Cardiomyopathy	It is recommended to not proceed with pregnancy for EF less than 30% [4]	A previous cardiac event is most predictive of an adverse event. Higher New York Heart Association functional class is associated with adverse cardiac events [5]	Adverse events include heart/failure and or ventricular tachycardia, aborted sudden cardiac death, atrial fibrillation, cerebrovascular accident or transient ischemic attack and death
Hypertrophic Cardiomyopathy	It is recommended to not proceed with pregnancy if the LVOT obstruction is greater than 30 mmHg [3]	Symptomatic at the time of pregnancy, diastolic dysfunction have history of arrhythmia or significant LVOT obstruction, ZAHARA or CARPREG score ≥1 [2]	Adverse cardiac events include dyspnea, heart failure, arrhythmia, angina, dizziness, syncope and rarely death
Arrhythmogenic Ventricular Cardiomyopathy	Symptomatic patients are advised to avoid pregnancy until symptoms are well controlled +/− ICD (if clinically appropriate)	CARPREG Score ≥1, recurrent uncontrolled arrhythmias, NYHA Class III or VI heart failure symptoms	Adverse cardiac symptoms are dizziness, dyspnea, palpitations, heart failure, occurrence of ventricular tachycardia (0–33%) and syncope [1]
Left Ventricular Non-Compaction	Pregnancy is not advised if patient is symptomatic	NYHA Class III or IV symptoms, Sustained Ventricular arrhythmias and enlarged left atrium	Ventricular tachycardia, thromboembolic phenomenon, heart failure
Restrictive Cardiomyopathy (primary and secondary)	Pregnancy is typically not advised in actively symptomatic patients	NYHA Class III or IV symptoms- Once patient has this diagnosis it is associated with a poor prognosis even with a near normal EF	Atrial fibrillation, heart failure, ascites, death, fetal loss

**Table 3:** Cardiovascular Drugs during Pregnancy.

Hypertension (Safe in Pregnancy)	Heart Failure (Safe in Pregnancy)	Pulmonary Hypertension (Safe in Pregnancy)	Arrhythmias (Safe in Pregnancy)	Anticoagulants/ Antiplatelets (Safe in Pregnancy)	Not safe or Limited Data in Pregnancy
B Oral alpha-methyl dopa * Hydrochlorothiazide *	B Bumetanide Torsemide Metolazone Dobutamine	B Sildenafil * Epoprostenol	B Lidocaine * Sotalol	B Enoxaparin * Argatroban Bivalirudin Fondaparinux Clopidogrel Prasugrel	Not safe D DOACs Atenolol Amiodarone (Can be used as a last resort) ACE-Inhibitors * Aldosterone antagonists Angiotensin receptor blockers Statins Dronaderone Dofetilide Ivabradine
C Labetalol * Clonidine Nifedipine * Amlodipine * Nitroglycerin Hydralazine * Isodorbide dinitrate Nitroprusside * Bisoprolol *	C Furosemide * Carvedilol Metoprolol * Isosorbide dinitrate Nitroglycerin Norepinephrine Hydralazine * Dopamine	C Iloprost Treprostinil	C Flecainide * (F) Digoxin * (F) Metoprolol * Propranolol * Labetalol * Sotalol (F) Adenosine * Verapamil Procainamide Quinidine * Atropine	C Unfractionated heparin * Aspirin (81 mg) * Ticagrelor	
				D Warfarin * (Safe after first trimester until 36 weeks gestation)	Limited Data Mexiletine Ibutilide Propafenone * Disopyramide Nadolol Diltiazem

Former Food & Drug Administration ABCD categories along with the indications are listed. * Safe in lactation. (F) = Can be used for Fetal Tachycardia.

**Table 4:** Roles of MDCCT members.

	Preconception	Pregnancy	Labor & Delivery	4th Trimester	Long Term
Cardioobstetrics *Team lead*		Δ o	Δ o	Δ o	
Cardiology *Cardiac Expertise*	Δ	Δ o	Δ	Δ o	Δ
Maternal-Fetal Medicine *Pregnancy Expertise*		Δ o	Δ o	Δ o	
Obstetrics/Gynecology *Maternal care* *Contraception*	*	*	*		
Neonatology *Fetus/Newborn*		o	o	o	
Anesthesiology *Anesthesia, Critical care*		Δ	Δ o		
Cardiology & Critical Care Subspecialists *Arrhythmias, heart failure, critical care*	*	Δ	Δ	o	o
Genetics *Risk assessment & counseling*	Δ				
Pharmacy *Pharmacology*		o	o	o	
Social Services *Communication, resources, connections*	+	+	+	+	
Hospitalist & Nursing *Hospital-based care*		+	+	+	

* Varies depending on risk status and institutional practices. + Particularly important care transitions. Δ Key activity: Risk assessment. o: Key activity: Management.

## Data Availability

Not applicable.
